# Integrated Analysis of Gene Expression Profiles Associated with Response of Platinum/Paclitaxel-Based Treatment in Epithelial Ovarian Cancer

**DOI:** 10.1371/journal.pone.0052745

**Published:** 2012-12-27

**Authors:** Yong Han, Hao Huang, Zhen Xiao, Wei Zhang, Yanfei Cao, Like Qu, Chengchao Shou

**Affiliations:** 1 Key Laboratory of Carcinogenesis and Translational Research (Ministry of Education), Department of Biochemistry and Molecular Biology, Peking University Cancer Hospital and Institute, Beijing, China; 2 Department of Gynecology, Wuhan University Renmin Hospital, Wuhan, Hubei, China; 3 Changzhi Medical College, Changzhi, Shanxi, China; Cedars-Sinai Medical Center, United States of America

## Abstract

**Purpose:**

This study aims to explore gene expression signatures and serum biomarkers to predict intrinsic chemoresistance in epithelial ovarian cancer (EOC).

**Patients and Methods:**

Gene expression profiling data of 322 high-grade EOC cases between 2009 and 2010 in The Cancer Genome Atlas project (TCGA) were used to develop and validate gene expression signatures that could discriminate different responses to first-line platinum/paclitaxel-based treatments. A gene regulation network was then built to further identify hub genes responsible for differential gene expression between the complete response (CR) group and the progressive disease (PD) group. Further, to find more robust serum biomarkers for clinical application, we integrated our gene signatures and gene signatures reported previously to identify secretory protein-encoding genes by searching the DAVID database. In the end, gene-drug interaction network was constructed by searching Comparative Toxicogenomics Database (CTD) and literature.

**Results:**

A 349-gene predictive model and an 18-gene model independent of key clinical features with high accuracy were developed for prediction of chemoresistance in EOC. Among them, ten important hub genes and six critical signaling pathways were identified to have important implications in chemotherapeutic response. Further, ten potential serum biomarkers were identified for predicting chemoresistance in EOC. Finally, we suggested some drugs for individualized treatment.

**Conclusion:**

We have developed the predictive models and serum biomarkers for platinum/paclitaxel response and established the new approach to discover potential serum biomarkers from gene expression profiles. The potential drugs that target hub genes are also suggested.

## Introduction

Epithelial ovarian cancer, which accounts for over 90% of all ovarian cancers, occurs most commonly in sixth and seventh decades of postmenopausal women and is a leading cause of cancer death among women in developed countries [1]. In the United States, there were approximately 21,990 new cases of ovarian cancer diagnosed and 15,460 deaths in 2011 [2]. Primary cytoreductive surgery followed by postoperative chemotherapy is considered the standard of care for advanced ovarian cancer [3]. First-line chemotherapy with platinum and paclitaxel agents is capable of achieving a complete response (CR) in the approximately 70% of patients with advanced disease [4]. However, about 30% patients do not respond to these drugs and even the patients who initially respond to first line chemotherapy frequently relapse and eventually become resistant to those agents.

How to predict chemotherapeutic resistance and even more importantly, how to reverse the resistance are clinically challenged. One of approaches is to identify predictive biomarkers especially those biomarkers that could be also therapeutic targets. Gene expression profiling technology was used to identify chemoresistance-related biomarkers [5]–[13]. However, to date, no gene expression signature has been proved to be sufficiently effective in predicting chemoresistance in clinical practice, which is largely due to inappropriate sample inclusion and/or small sample size used in the studies.

To address this challenge, we critically selected and assessed 322 serous ovarian cancer patients only with CR or progressive disease (PD) to platinum/paclitaxel-based therapy from The Cancer Genome Atlas (TCGA) project to identify gene expression signatures associated with chemoresistance. By using supervised principal components method, a 349-gene predictive model and an 18-gene de-correlated model independent of patient age, stage, debulking status or tumor stages were developed for chemoresistance prediction. Further, to identify serum chemotherapeutic biomarkers for more practically clinical application, we combined our 322-gene expression profile and four previous findings, which were selected based on the strict criteria of gene expression profile of the epithelial ovarian cancer, validation, appropriate sample size, and treatment response prediction of first line chemotherapy. We found ten serum biomarkers that have predictive value for primary response to first-line chemotherapy. In the end, several drugs that could target hub genes in our models were suggested. Our results provide a platform for the selection of the most suitable drugs for a better treatment outcome of those patients resistant to platinum/paclitaxel-based chemotherapy.

## Patients and Methods

### Ethics Statement

We are free to use ovarian cancer data in TCGA by meeting its freedom-to-publish criteria: A marker paper has been published on that tumor type. The Research Ethics Committee of Peking University Cancer Hospital & Institute waived the requirement for ethical approval of this analysis because the registry is a de-identified database. Written consents were obtained from all alive patients.

### Patients and Tissue Samples

A total of 322 patients with high-grade serous ovarian cancer were carefully selected from TCGA database (National Cancer Institute. The cancer genome atlas data portal. http://tcga-data.nci.nih.gov/tcga/findArchives.htm. Accessed September 1, 2011). Detailed information of the selected patients including age at diagnosis, tumor stage, grade and debulking status are listed in Table 1. All ovarian cancer specimens' information and clinical definitions were previously described [14]. All selected patients received a first-line platinum/paclitaxel-based treatment except that four patients' treatment regimen was unknown. The 322 samples were randomly divided into training (n = 200) and testing sets (n = 122). In the training set, 177 of 200 patients demonstrated CR and 23 of 200 patients demonstrated PD to primary platinum/paclitaxel-based therapy after surgery. In the testing set, 110 of 122 patients had CR and 12 of 122 patients had PD to platinum/paclitaxel-based treatment.

**Table 1:** Clinicopathological Characteristics of Ovarian Cancer Patients.

Characteristics	Clinical Complete Responders (*n* = 287)	Progressive Desease (*n* = 35)	*p*-value
Mean age, years	58.76655	59.91429	0.574^a^
Stage(FIGO), No. of patients			0.065^b^
II	20	0	
III	228	26	
IV	39	9	
Grade, No. of patients			0.484^b^
2	35	6	
3	251	29	
4	1	0	
Surgical debulking, No.of patients			0.001^b^
None	70	2	
≤1 cm	138	16	
1 cm∼2 cm	13	6	
>2 cm	38	10	
Unknown	28	1	
First-line chemotherapy			1^b^
Platinum-based Taxane (paclitaxel or docetaxel)	283	35	
Unknown	4	0	

Abbreviations: FIGO = Fe'de'ration Internationale de Gyne'cologie et Obste'trique;

a Mann-Whitney test.

b Fisher's exact test.

### Selection of Related Studies Published Previously

In order to find previous studies closely related to our study, we searched online databases from 2005∼2011 with strict criteria: the same cancer subtype, validation, appropriate sample size, and treatment response prediction of first line chemotherapy. Four studies were identified [6], [8], [10], [11] and their detailed information was listed in Table 2. For the validation of our signature genes, 3 datasets [15]–[17] from NCBI GEO database [18] were downloaded. These 3 datasets are all gene expression profiles of chemoresistant ovarian cancer cell lines ‘A2780-resistant’ and parental cell line ‘A2870’, which were independently generated by 3 different groups. There are 5, 3 and 6 replicates in datasets GSE15372, GSE28646, and GSE33482 respectively. Genes that are closely related to platinum/paclitaxel treatment response are also searched on the CTD database.

**Table 2:** Selection of the previously published gene signatures associated with response to platinum/Paclitaxel-based treatment (2005 to 2011).

Publication	Platform	No.of genes	Samples investigated	Journal
Dressman et al, 2007	Affymetrix Human U133A GeneChip	1388	119 advanced-stage serous ovarian cancers	*J Clin Oncol*
Helleman et al, 2006	18K cDNA microarrays	68	96 primary ovarian adenocarcinoma(mainly serous)	*Int J Cancer*
Jazaeri et al, 2005	Combined two cDNA microarrays contained 32,448 and 7,585 features each	85	21primary chemosensitive tumors and 24 primary chemoresistant tumors(mainly serous)	*Clin Cancer Res*
Ju et al, 2009	Affymetrix Human U133A GeneChip	100	5 primary chemosensitive tumors and 8 primary chemoresistant tumors	*Oncol Res*

### Gene Expression Profiling Analysis

Gene expression profiling data (level 3) of 322 serous ovarian cancer samples were obtained from the TCGA Data Portal. The profiling of all the samples was performed on the Human U133A Gene Chip (Affymetrix, Santa Clara, CA).

### Bioinformatics and Statistical Analysis

The supervised principal components method was employed for generating a general predictive gene model and a gene model that is independent of key clinical features including age, stage, debulking status, and grade (de-correlated model). The analysis above was conducted using superpc package [19] in R 2.14.0 (R Foundation for Statistical Computing [http://www.r-project.org/]). The differentially expressed genes in 3 GEO dataset were also computed in R. We used ChEA for transcription factor analysis [20], DAVID and Clone/Gene ID Converter for gene annotation [21], [22], and GATHER for pathway enrichment analysis [23]. GNCpro (http://gncpro.sabiosciences.com/gncpro/gncpro.php), C3NET package [24] in R 2.14.0, MiMI plugin [25], and GeneMANIA [26] plugin in Cytoscape [27] were employed to explore and plot gene-gene interaction network and transcription factor network as well as top ten genes' interaction network and gene-drug interaction network.

Standard statistical tests were used to analyze the clinical and gene expression profiling data, including the χ^2^ test, fisher exact test and independent samples *t*-test. Significance was defined as a *p* value of less than 0.05. Benjamini-Hochberg multiple testing correction was used to estimate the false discovery rate in the pathway analysis [28]. Receiver operating characteristic (ROC) curve and Area Under the Curve (AUC) were used for signature predictability evaluation. Analyses were primarily performed using R and SPSS version 18 (SPSS Inc, Chicago, Illinois).

## Results

### Development of Predictive Models Associated With Chemotherapeutic Response

**Figure 1:**
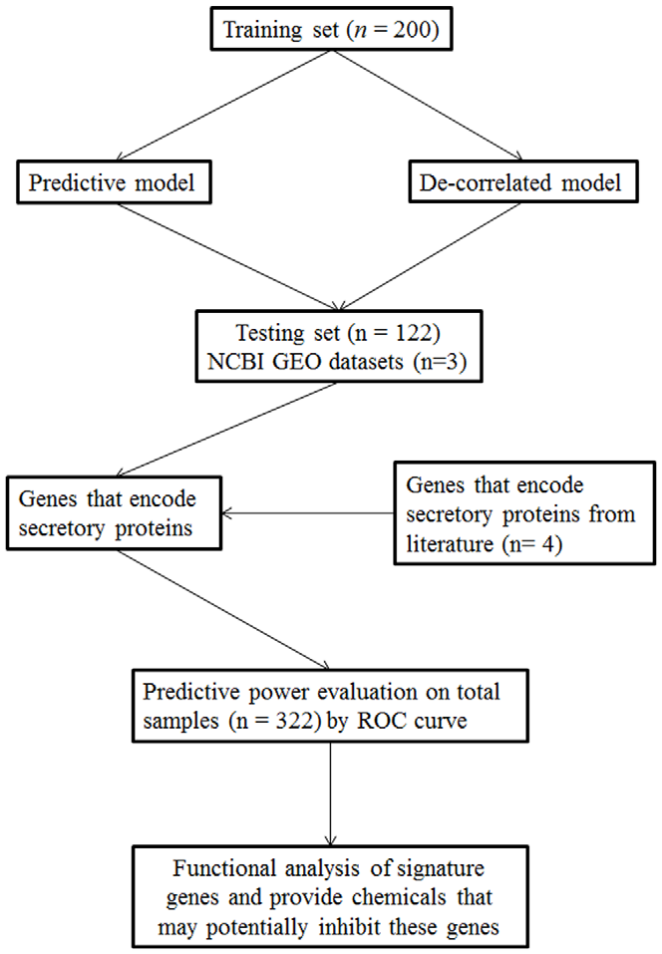
Work flow of the study design. The 322 high-grade serous ovarian cancer cases were randomly divided in the training set (200 samples) and the testing set (122 samples). The training set was used to generate the predictive model and de-correlated model that is independent of key clinical features. Then these two models were validated using the testing set. Next we used 3 datasets from GEO database to validate signature genes in our findings. To explore potential biomarkers in serum, we combined signature genes in these two models with genes previously reported in four previous studies and queried these genes in DAVID database. Seventy-seven genes encoding secretory proteins were identified (Table S3). The predictability of those genes for chemotherapeutic response was then tested individually using the data from all 322 samples. Finally, we performed a functional analysis on those signature genes and suggested some drugs that could target the hub genes in our findings.

To identify a gene expression signature that predicts response to chemotherapy and thus help determine the most appropriate regimen for personalized treatment, a 349-gene predictive model and an 18-gene de-correlated model were developed from the training set using superpc package in Bioconductor (Fig. 1). Specifically, first we computed the univariate regression score of each feature (12042 genes) in regard to patient's treatment outcome (CR or PD). Then we performed a 10 fold cross-validation to find out the best threshold and to form a reduced data matrix consisting of only those features whose score exceeds a threshold (in our case, the best threshold is 1.26). Then we performed principal component analysis to find out the most significant gene set for prediction of treatment response. It turns out the first principal component containing 349 features is the best (*p* = 0.025). In order to keep the strongest power in prediction of response, we didn't do the shrinkage. Finally, the most significant principal component in a regression model was used to predict the treatment outcome. Similarly, in case of developing de-correlated model, first we fitted a linear model to key clinical features (age, stages, debulking status, and grade) as competing predictors, and then we replaced these features by the residual from this fit. In the superpc model building process, these ‘de-correlated’ features are used to explicitly look for predictors which are independent of key clinical features. We chose the threshold 1.85 and the first principal component contains 18 features with p = 0.001. Since 18-gene model is a small gene set, we didn't do the shrinkage either.

**Figure 2:**
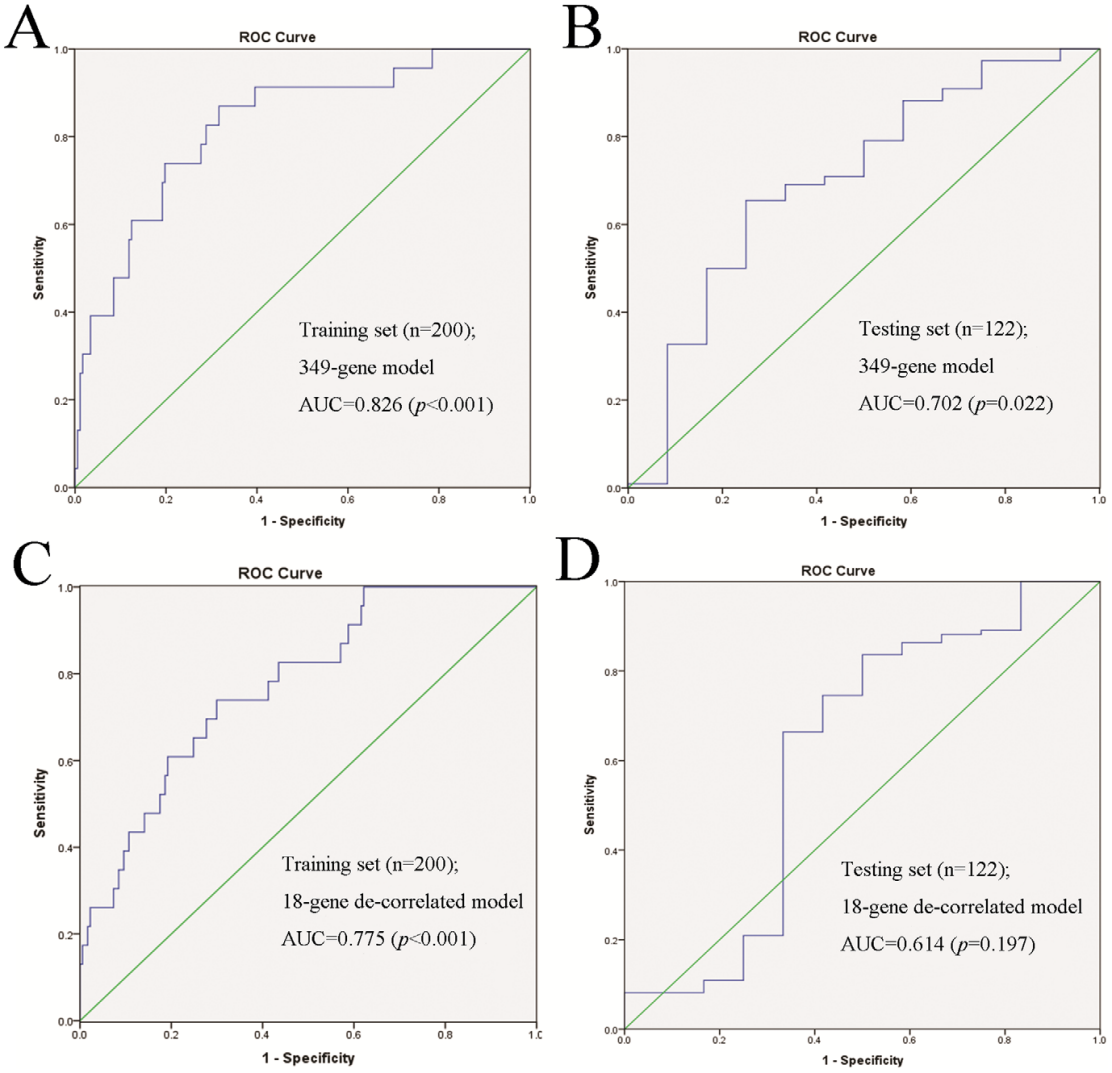
ROC curves of the two predictive models in the training set and the testing set. (A) ROC curve of the 349-gene predictive model in training set (200 samples, AUC = 0.826; *p<*0.001. (B) ROC curve of the 349-gene predictive model in the testing set (122 samples, AUC = 0.702; *p* = 0.022). (C) ROC curve of the 18-gene de-correlated predictive model in the training set (200 samples, AUC = 0.775; *p*<0.001. (D) ROC curve of the 18-gene de-correlated predictive model in the testing set (122 samples, AUC = 0.614; *p* = 0.197).

As shown in Fig. 2, the 349-gene signature had an AUC = 0.826 (*p*<0.001) in the training set (Fig. 2A) and AUC = 0.702 (*p* = 0.022) in the testing set (Fig. 2B). The 18-gene de-correlated signature had an AUC = 0.775 (*p*<0.001) in the training set (Fig. 2C) and AUC = 0.614 (*p* = 0.197) in the testing set (Fig. 2D). In the 349-gene model, 30 most weighted genes were listed in Table 3 (We determined the top 30 genes by the rank of importance-score of each gene in Fig. S6, which was computed for each gene equal its correlation with the supervised principal component predictor. See Table S1 for all genes' information in this model) and 18 genes in the 18-gene de-correlated model were listed in Table 4 (important-score of each gene is listed in Fig. S7).

**Table 3:** Top 30 weighted genes in 349-gene signature.

Gene Symbol	Description	Function
**C6orf120**	Chromosome 6 open reading frame 120	Secreted, signal, extracellular region
**BLMH**	Bleomycin hydrolase	response to toxin, response to drug
**ACTR6**	ARP6 actin-related protein 6 homolog (yeast)	Actin/actin-like
**USP21**	Ubiquitin specific peptidase 21	positive regulation of transcription, chromatin modification
**NIT1**	Nitrilase 1	nitrilase activity, hydrolase activity
**VPS72**	Vacuolar protein sorting 72 homolog (S. cerevisiae)	negative regulation of gene expression, chromatin regulator
**KIAA0859**	KIAA0859	methyltransferase, tumor promoter
**GTF2H5**	General transcription factor IIH, polypeptide 5	DNA repair,response to DNA damage stimulus
**PIGC**	Phosphatidylinositol glycan anchor biosynthesis, class C	protein amino acid lipidation, GPI anchor metabolic process
**C1orf25**	Chromosome 1 open reading frame 25	tRNA (guanine) methyltransferase activity, ion binding
**TBP**	TATA box binding protein	transcription regulation,CARM1 and Regulation of the Estrogen Receptor
**NCSTN**	Nicastrin	positive regulation of apoptosis, Notch signaling pathway
**SF3B4**	Splicing factor 3b, subunit 4, 49 kDa	RNA splicing factor activity, transesterification mechanism
**SCAMP3**	Secretory carrier membrane protein 3	response to extracellular stimulus, protein transport
**MTX1**	Metaxin 1	establishment of protein localization, intracellular transport
**C1orf27**	Chromosome 1 open reading frame 27	oxidation reduction, metal ion binding
**RHOT1**	Ras homolog gene family, member T1	microtubule-based transport, programmed cell death,small GTPase mediated signal transduction
**ZNF200**	Zinc finger protein 200	regulation of transcription, transition metal ion binding
**SCNM1**	sodium channel modifier 1	mRNA processing, transition metal ion binding
**DDX23**	DEAD (Asp-Glu-Ala-Asp) box polypeptide 23	mrna processing, cellular macromolecular complex assembly
**SSR2**	Signal sequence receptor, beta (translocon-associated protein beta)	establishment of protein localization, intracellular transport,signal sequence binding
**ENSA**	Endosulfine alpha	response to extracellular stimulus, ion channel inhibitor activity
**PDCD2**	Programmed cell death 2	apoptosis, dna-binding, metal-binding
**TIMM17B**	Translocase of inner mitochondrial membrane 17 homolog B (yeast)	protein localization
**NFS1**	NFS1 nitrogen fixation 1 homolog (S. cerevisiae)	sulfurtransferase activity,cysteine metabolic process
**GNPDA1**	Glucosamine-6-phosphate deaminase 1	alcohol catabolic process, amino sugar catabolic process
**NKIRAS2**	NFKB inhibitor interacting Ras-like 2	I-kappaB kinase/NF-kappaB cascade,small GTPase mediated signal transduction
**ENOPH1**	Enolase-phosphatase 1	Cysteine and methionine metabolism
**TTC31**	Tetratricopeptide repeat domain 31	unknown
**NUDT9**	Nudix (nucleoside diphosphate linked moiety X)-type motif 9	purine nucleotide metabolic process, ion transport

**Table 4:** The 18 signatured genes in the 18-gene de-correlated model.

Gene Symbol	Gene Name	Function
AFF1	AF4/FMR2 family, member 1	positive regulation of gene expression, Proto-oncogene
AFM	afamin	serum transport proteins
CLCA4	chloride channel accessory 4	calcium-activated chloride channel
CXXC4	CXXC finger 4	chemotherapy resistance, metal-binding
ESR2	estrogen receptor 2 (ER beta)	negative regulation of apoptosis,metal-binding, dna-binding
HSD17B2	hydroxysteroid (17-beta) dehydrogenase 2	response to chemical stimulus
LMO1	LIM domain only 1 (rhombotin 1)	cell proliferation,apoptosis regulation, Proto-oncogene, metal-binding
MVK	mevalonate kinase	isoprenoid and sterol synthesis,atp-binding
OPCML	opioid binding protein/cell adhesion molecule-like	tumor suppressor, cell adhesion
PAPPA	PAPPA antisense RNA (non-protein coding); pregnancy-associated plasma protein A, pappalysin 1	wound healing and angiogenesis, metal-binding
PDCD1LG2	programmed cell death 1 ligand 2	regulation of immune system process
PSMD4	proteasome (prosome, macropain) 26S subunit, non-ATPase, 4	mitotic cell cycle, metal-binding, rna-binding, atp-binding
RNASEL	ribonuclease L (2′,5′-oligoisoadenylate synthetase-dependent)	tumor suppressor, metal binding
SEMA4F	sema domain, immunoglobulin domain (Ig), transmembrane domain (TM) and short cytoplasmic domain, (semaphorin) 4F	regulation of cell growth
SLC17A7	solute carrier family 17 (sodium-dependent inorganic phosphate cotransporter), member 7	ion transport, cell junction
TNFSF11	tumor necrosis factor (ligand) superfamily, member 11	regulation of cell apoptosis
TRIM15	tripartite motif-containing 15	metal-binding
ZP2	zona pellucida glycoprotein 2 (sperm receptor)	cell-cell recognition, microenvironment

Based on these results, the 349-gene model had high sensitivity and specificity in both the training set and the testing set. The 18-gene de-correlated model had good sensitivity and specificity in the training set, but relatively low sensitivity and specificity in the testing set.

### Functional Analysis of the Signature Genes from the Two Predictive Models

**Figure 3:**
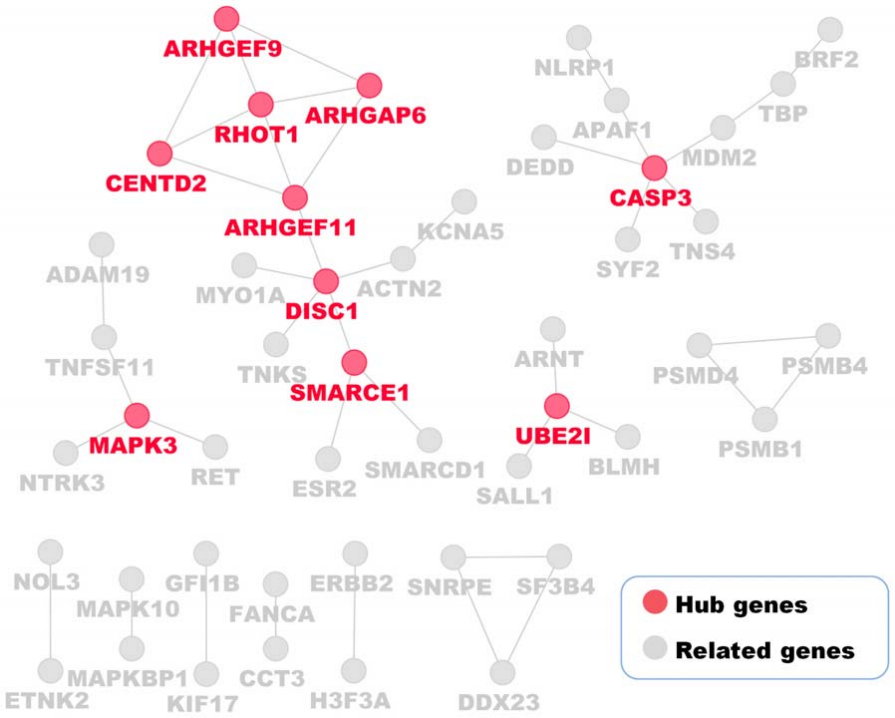
Ten hub genes in the 349-gene signature. Genes that interact with at least three other genes were selected, among which UBE2I, CASP3 and MAPK3 are important molecules that are involved in ovarian cancer progression or chemoresistance. Detailed information of these ten hub genes are listed in Table 4.

To understand the biological roles of the signature genes from the 349-gene predictive model and the 18-gene de-correlated model involved in chemoresistance, we performed three types of analyses. First, we conducted a gene-gene interaction network analysis to identify hub genes in the 349-gene model using MiMI plugin in Cytoscape (Fig. 3). By defining hub genes as genes that interact with at least three other genes, ten hub genes were identified (Table 5), of which UBE2I (Ubiquitin-conjugating enzyme E2I) [29], [30], CASP3 (Caspase 3, apoptosis-related cysteine peptidase) [31] and MAPK3 (Mitogen-activated protein kinase 3) [32], [33] are closely associated with platinum/paclitaxel-based chemotherapeutic response.

**Table 5:** Ten hub genes in the 349-gene signature.

Gene Symbol	Description	Function
**UBE2I**	Ubiquitin-conjugating enzyme E2I	mitotic cell cycle, negative regulation of gene expression
**SMARCE1**	SWI/SNF related, matrix associated, actin dependent regulator of chromatin, subfamily e, member 1	negative regulation of transcription, chromatin modification
**CASP3**	Caspase 3, apoptosis-related cysteine peptidase	response to tumor necrosis factor, regulation of cell proliferation
**DISC1**	Disrupted in schizophrenia 1	microtubule organizing center
**ARHGEF11**	Rho guanine nucleotide exchange factor (GEF) 11	regulation of cell growth, regulation of apoptosis
**CENTD2**	Centaurin, delta 2	promote tumor survival
**RHOT1**	Ras homolog gene family, member T1	microtubule-based transport, programmed cell death,small GTPase mediated signal transduction
**ARHGAP6**	Rho GTPase activating protein 6	negative regulation of cell-matrix adhesion, small GTPase mediated signal transduction
**ARHGEF9**	Cdc42 guanine nucleotide exchange factor (GEF) 9	induction of apoptosis by extracellular signals, regulation of Ras protein signal transduction
**MAPK3**	Mitogen-activated protein kinase 3	Ras protein signal transduction, cell cycle

Considering that most changes in gene expression are regulated by upstream regulatory transcription factors and/or signaling genes, we then searched on ChEA for transcription factors that could regulate the 349 genes. Twenty nine transcription factors with statistical significance (*p*<0.01) were found (Table S2). Based on different interaction types, we constructed an interaction network of these transcription factors (Fig. S5).and performed a pathway enrichment analysis using these factors. We found that six pathways were most related to eight of the 29 transcription factors, including MAPK signaling pathway (*p* = 0.0007), TGF-beta signaling pathway (*p* = 0.001), cell cycle (*p* = 0.001), Wnt signaling pathway (*p* = 0.003), focal adhesion (*p* = 0.007), and cell proliferation (*p* = 0.02). These pathways may play important roles in chemoresistance in ovarian cancer. Detailed information of the transcription factors are listed in Table 6 and Table S2.

**Table 6:** Six enriched pathways of 29 transcription factors derived from the 349-gene model.

Annotation	Transcription Factors	*p*-value
MAPK signaling pathway	ELK1 JUN MYC	0.0007
TGF-beta signaling pathway	E2F4 MYC	0.001
Cell cycle	E2F1 E2F4	0.001
Wnt signaling pathway	JUN MYC	0.003
Focal adhesion	ELK1 JUN	0.007
Cell proliferation	AR E2F1 E2F4 ESR1 ETS1 MYC	0.02

The gene-gene interaction network of the 18-gene model was built by C3NET in Bioconductor (Fig. S6A), in which 17 genes have physical interactions with the other genes. To gain a more complete picture of the 18 genes and their interacting neighbors, we constructed a network using GNCpro (Fig. S6B), in which 11 of 18 genes have interactions with the other genes and PAPPA, TNFSF11, and ESR2 are important hub genes in the 18-gene model. As shown in Fig. S6B, TNFSF11 up-regulates critical transcription factors such as JUN, SRC and AKT1, ESR2 has physical interactions with SP1, AKT1 and SRC, and PAPPA modifies IGFBP4 [34]–[40].

### Hunting for Potential Serum Biomarkers for Chemotherapeutic Response

Since serum biomarker is most conveniently detected in clinics, we sought to set up a new way to identify potential serum biomarkers for chemotherapeutic response from gene expression profiles by targeting genes encoding secretory proteins. We integrated genes in 349-gene model and 18-gene model and those genes from the four previous studies and searched for genes encoding the secretory proteins in DAVID database (Fig. 1). As a result, 77 genes were identified to encode the secretory proteins that could be secreted into the serum (Table S3). We then tested the predictive values of these genes individually for chemotherapeutic response using the 322 gene expression profiling data and computed the AUC value of these genes (Table S4). Top ten genes with highest AUC values (Table 7) were found to have the ability to discriminate the CR group from the PD group (*p*<0.05), of which AFM has been reported to be an independent serum biomarker of CA125 for the prediction of ovarian cancer progression by comparative proteomics analysis [41], [42].

**Table 7:** Area Under the Curve (AUC) of Top Ten genes (*p*<0.05) that encode secretory proteins.

Gene	AUC	Std. Error^a^	Asymptotic Sig.^b^	Asymptotic 95% Confidence Interval	
				Lower Bound	Upper Bound
CLPS	0.637	0.041	0.008	0.556	0.718
C1orf56	0.636	0.048	0.009	0.542	0.730
AFM	0.630	0.055	0.012	0.523	0.737
GIP	0.618	0.052	0.022	0.515	0.721
PRG4	0.618	0.052	0.023	0.515	0.721
CPA2	0.617	0.046	0.024	0.527	0.707
FOLR1	0.613	0.047	0.029	0.521	0.705
IL1RL1	0.608	0.046	0.037	0.518	0.699
COMP	0.604	0.055	0.045	0.496	0.711
C6orf120	0.602	0.050	0.048	0.504	0.700

Abbreviations: Std.: standard. Sig: significance.

a Under the nonparametric assumption;

b Null hypothesis: true area = 0.5.

### Functional Analysis of Potential Serum Therapeutic Biomarkers

**Figure 4:**
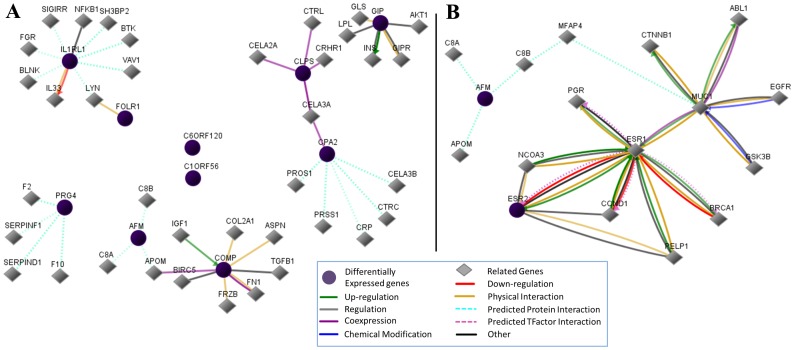
Hub genes and gene-gene interaction networks of top ten secretory protein-encoding genes. (A) Hub genes and neighboring genes of top ten secretory protein-encoding genes. (B) AFM was exemplified to show potential mechanisms of the top ten secretory protein-encoding genes probably involving in chemoresistance.

To further investigate the roles of the top ten serum biomarkers in chemotherapeutic response, we constructed a gene/protein interaction network using GNCpro. As shown in Fig. 4A, IL1RL1, PRG4, AFM, GIP and COMP appeared to be critical hub genes since they could interact with the genes known to be involved in chemoresistance. For example, AFM seems to interact indirectly with MUC1, ESR1, and BRCA1 that are known to contribute to the resistance to the platinum/paclitaxel-based treatment (Fig. 4B).

### Further validation of genes in our signature, hub genes and potential serum biomarkers

**Figure 5:**
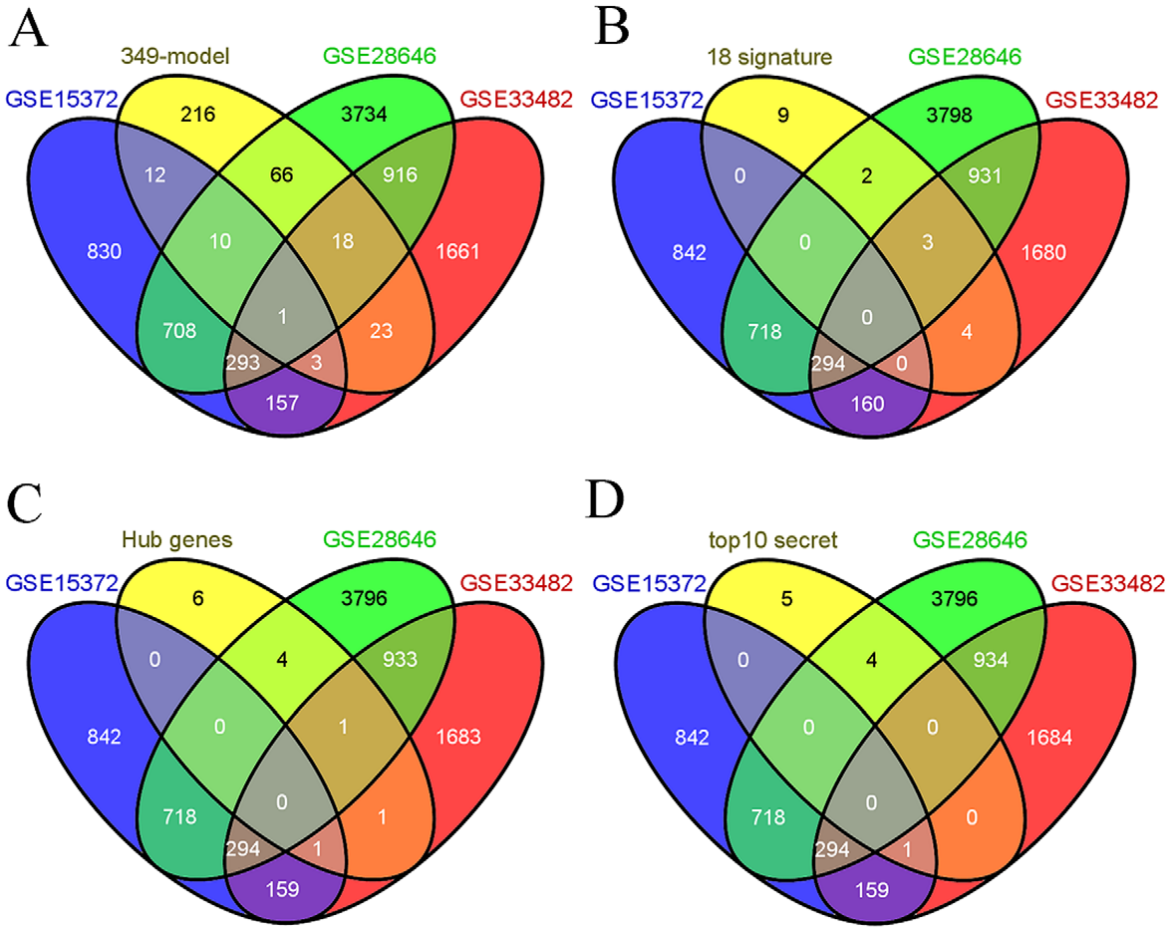
Venn diagram showing the overlap between our signatures genes and 3 external datasets from NCBI GEO database. The Venn diagram shows how much genes in the 349-gene model (A), 18-gene model (B), hub genes (C), and top 10 serum biomarkers (D) are overlapped with 3 external datasets GSE15372, GSE28646 and GSE33482.

We further validated genes in our signature, hub genes and potential serum biomarkers in 3 more ways. First, we use 3 different datasets (GSE15372, GSE28646 and GSE33482) from NCBI GEO database to validate our data. These 3 datasets are all gene expression profiles of chemoresistant ovarian cancer cell lines ‘A2780-resistant’ and parental cell line ‘A2870’, which were generated by 3 different groups. There are 5, 3 and 6 replicates in datasets GSE15372, GSE28646 and GSE33482, respectively. Differentially expressed genes in these 3 dataset were computed and displayed in Table S8. We use the Venn diagram to show the overlap between our signature genes and those differentially expressed genes (Fig. 5). 133 genes of 349-gene model, 9 genes of 18-gene model, 7 of 13 hub genes and 5 of 10 potential serum biomarkers are overlapped with differentially expressed genes from those 3 datasets (Fig. 5A, 5B, 5C, and 5D, respectively).

Meanwhile, we searched CTD database for genes that could associated with sensitivity to platinum/paclitaxel-based drugs (Table S5), then the overlap between our signature genes and searched results was also presented (Fig. S4C). 30 of 349-gene model, 3 of 18-gene model and 4 of 13 hub genes are overlapped with searched results.

In addition, we presented the overlap between our signature genes and those signature genes from 4 literatures (Fig. S3 and S4B). 16 genes of 349-gene model, 0 genes of 18-gene model, 1 of 13 hub genes, and 2 of 10 potential serum biomarkers are overlapped with gene signatures from 4 previous publications (Table S9). However, as we can see from Figure S4D, signature genes from literatures in Table 2 also shown little overlap among them. This may due to their relatively small sample size, different standard of sample selection, or different methods to develop predictive models.

### Construction of Gene-drug interaction network and gene targeting drug suggestion

**Figure 6:**
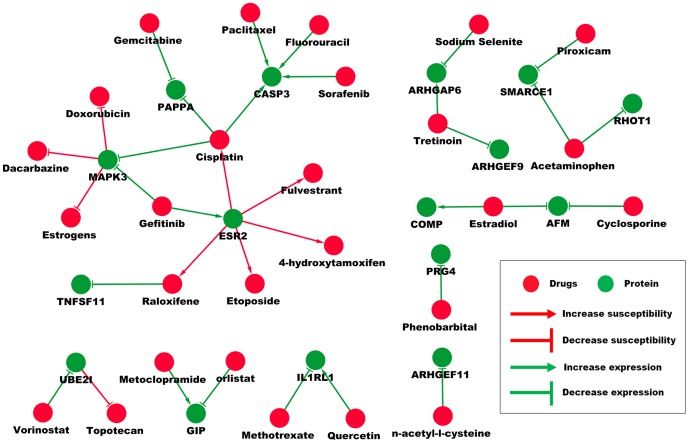
Hub gene-drug interaction network. The hub gene-drug interaction network shows us how these genes and drugs could interact with each other. For example, ESR2 could increase the patient's susceptibility to Cisplatin, Etoposide and Raloxifene, while Gefitinib could increase the expression of ESR2.

Since we already got key transcription factors and hub genes, we might want to know which drugs could target these genes in order to reverse the resistance to platinum/paclitaxel-based treatment. By searching on The CTD and NCBI Pubmed Database, several drugs and specific inhibitors were found to interact with our key transcription factors and hub genes. By combining these results, we build a key transcription factor-drug interaction network (Fig. S7) and a hub gene-chemical interaction network (Fig. 6), which not only show us which chemicals can inhibit these key transcription factors and hub genes, but also tell us how these genes could increase or decrease the susceptibility of chemotherapeutic drugs. For example, ESR2 could increase the patient's susceptibility to Cisplatin, Etoposide and Raloxifene, while Gefitinib could increase the expression of ESR2. MAPK3 could decrease the patient's susceptibility to Doxorubicin, Dacarbazine and Estrogens, while Gefitinib and Cisplatin could decrease the expression of MAPK3, which suggested Gefitinib might be a good drug for platinum/paclitaxel-resistant patients.

## Discussion

Prediction of chemotherapeutic response is always a challenging clinical task. Many efforts have been conducted to find gene expression signatures to discriminate different responders using the high-throughput technology. However, none of those gene signatures is formally used in clinics. Possible reasons might be lacking of critical sample selection or small sample size. Our study and 4 previous publications listed in Table 2 are all aimed to find out gene signatures for predicting platinum-based treatment outcome of serous ovarian cancer. And there are several genes overlapped between our signature genes and those signature genes from previous publications. However, there are several differences among ours and those 4 publications. The statistical methods for developing models we use (Dressman et al. shotgun stochastic search [10], Ju et al. manually select top differentially expressed genes [11], Helleman et al. BRB & SAM [8], and Jazaeri et al. BRB [6]), sample size, and selection standard are not the same. Our model size (349-gene signature) is more appropriate compared with Dressman et al. (1704 probes representing 1388 genes). Although Ju et al., Helleman et al. and Jazaeri et al have smaller model size (100, 68, and 85 respectively), they either had small training set or merely used top differentially expressed genes as predictors.

In conclusion, our signatures are novel compared to the 4 previous publications (Table 2) in 4 aspects: 1) We have a bigger sample size (322 compared with 119, 96, 45 and 13), which are more convincible in developing predictive models. 2) We have a better sample selection (just using CR and PD samples and mainly focusing on platinum/paclitaxel resistance). 3) In order to find out which genes are truly related to treatment outcomes and to exclude the potential bias of those key clinical features, we developed a de-correlated model which was novel. 4) We employed supervised component analysis to develop 349-gene signature and 18-gene signature, which is different from that just using highest differentially expressed genes (Ju et al.).

To further elucidate the biological contribution of those signature genes to treatment outcomes, we built the regulatory networks to identify critical hub genes and signaling pathways differentially present in the CR and PD groups. Among the ten hub genes identified in the 349-gene model, UBE2I is correlated with histological subtypes of EOC [43], CASP3 is the most important marker of apoptosis [44], and MAPK3 plays a crucial role in EOC progression. The other genes (SMARCE1, DISC1, CENTD2, RHOT1, ARHGAP6, ARHGEF9 and ARHGEF11) are also involved in cancer progression or chemoresistance [45]–[49].

Since gene expression profiles are noisy and it is hard to find most significant pathways by doing the pathway enrichment directly, we set up a new strategy to solve this problem. Our strategy is based on two aspects: 1) most gene expression changes are regulated by transcription factors and 2) the action of transcription factors is relatively less noisy. By this approach, we found that the genes in the 349-gene model are regulated by 29 transcription factors that are enriched in the six critical pathways, including MAPK, TGF beta, Wnt, cell cycle, Focal adhesion, and cell proliferation signaling pathways. The association of these pathways with the response to chemotherapy or cancer progression has been reported in previous studies [50]–[58]. The hub genes, transcription factors and critical signaling pathways we identified could be potential targets for drug design after further validation.

One important feature of an ideal biomarker is easy to detect. We thus developed a new approach to screen chemotherapeutic biomarkers that could be detected in the serum. We found ten genes encoding secretory proteins that have the ability to separate CR from PD and thus could be potential serum biomarkers for predicting the response to the platinum/paclitaxel-based treatment in EOC. AFM identified in the study was reported to be an independent diagnostic marker of CA125 [42], which partly supports our strategy and findings. Since CA125 is the conventional biomarker for ovarian cancer progression and chemotherapeutic response, the addition of AFM to CA125 could thus improve the prognostic power in EOC.

The construction of gene-drug interaction network gave us more hints on how to choose the right drugs for individualized treatment. As shown in results section, Gefitinib might be an appropriate drug for the treatment of platinum/paclitaxel-resistant patients by decreasing the expression of MAPK3 and increasing the expression of ESR2.

Although our findings are encouraging, there are still some questions unanswered. For instance, experimental validations are still needed to explore the specific roles of those hub genes, transcription factors and signaling pathways in chemoresistance using ovarian cancer cell lines and animal. In addition, we need to further test those serum biomarkers using serum samples of ovarian cancer patients.

Summarily, we developed two predictive models that yield insights into the molecular mechanisms of chemoresistance. Based on the models, we built an upstream regulatory network in which several critical transcription factors and signaling pathways may play crucial roles in chemoresistance in EOC. Further, by integrating with published findings, we found ten potential serum biomarkers that could be used in clinical practice. In addition, gene-drug interaction network was constructed, which not only shows us which drugs can inhibit these key transcription factors and hub genes, but also tell us how these genes could increase or decrease the susceptibility of chemotherapeutic drugs. This is a good beginning for us to select the most suitable drugs for a better treatment outcome of those patients resistant to platinum/paclitaxel-based chemotherapy.

## Supporting Information

Table S1 **Gene list of the 349-gene signature.**
(XLSX)

Table S2 **Potential Transcriptional Factors that regulate genes in the 349-gene signature.**
(XLSX)

Table S3 **77 genes that encode secretory proteins related with platinum/paclitaxel-based treatment(integrated our results with 4 previous studies).**
(XLSX)

Table S4 **Area Under the Curve of genes that encode secretory proteins.**
(XLSX)

Table S5 **Genes interact with platinum or paclitaxel searched from CTD database.**
(XLSX)

Table S6 **Importance-scores of 349 genes in predictive regression model.**
(XLSX)

Table S7 **Importance-scores of 18 genes in de-correlated model.**
(XLSX)

Table S8 **Differentially expressed genes in GSE15372 & GSE33482 & GSE28646.**
(XLSX)

Table S9 **Signature genes from 4 previous publications which were listed in** **Table 2** **.**
(XLSX)

Figure S1 **Heat map of 349-gene signature against 322 patients.** This diagram shows the heat map of 349-gene signature against 322 patients, in which rows represent different genes in 349- gene signature and columns represent different patients. The blue bar above the heat map represents CR and green bar represents PD.
(TIFF)

Figure S2 **Heat map of 18-gene signature against 322 patients.** This diagram shows the heat map of 18-gene signature against 322 patients, where rows represent different genes in 18-gene signature and columns represent different patients. The blue bar above the heat map represents CR and green bar represents PD.
(TIFF)

Figure S3 **The Venn diagram showing the overlap between our signatures and genes from previous publications.** The Venn diagram shows how much genes in the 349-gene model (A), 18-gene model (B), hub genes (C), top 10 serum biomarkers (D) are overlapped with 3 previous publications (Dressman et al., Ju et al. and Jazaeri et al.).
(TIF)

Figure S4 **The Venn diagram showing the overlap among our signatures and other datasets & publications.** (A) The Venn diagram shows genes in 18-gene signature are all belong to genes in 349-gene signature. (B) The Venn diagram shows there are no overlap between genes from Helleman et al. and genes in our findings (349-gene signature, 18-gene signature and hub genes).
(TIF)

Figure S5 **Transcription factor Interaction network and enriched pathways derived from the 349-gene model.** Eight transcription factors circled in dot yellow line are enriched in TGF-beta, MAPK and Wnt signaling pathway (red arrow).
(TIF)

Figure S6 **Gene-gene interaction network of 17 genes in the 18-gene model and Hub gene interaction network of 18 signature genes.** (A) Gene-gene interaction network of 17 in the 18-gene model analyzed by C3NET. (B) Hub genes and neighboring genes of the 18 signature genes.
(TIF)

Figure S7 **Transcription factor- drug Interaction network.** This diagram shows how Transcription factors and drug are interacted. For example, MYC could decrease patient's susceptibility to Cisplatin, Fluorouracil and Doxorubicin, whereas 10058-F4 could decrease the expression of MYC.
(TIF)
