# Supplementary figures and images for: Integrated Analysis of Gene Expression Profiles Associated with Response of Platinum/Paclitaxel-Based Treatment in Epithelial Ovarian Cancer
Source: PLoS One. 2012 Dec 27;7(12):e52745. doi: 10.1371/journal.pone.0052745 (PMC3531383; doi:10.1371/journal.pone.0052745)

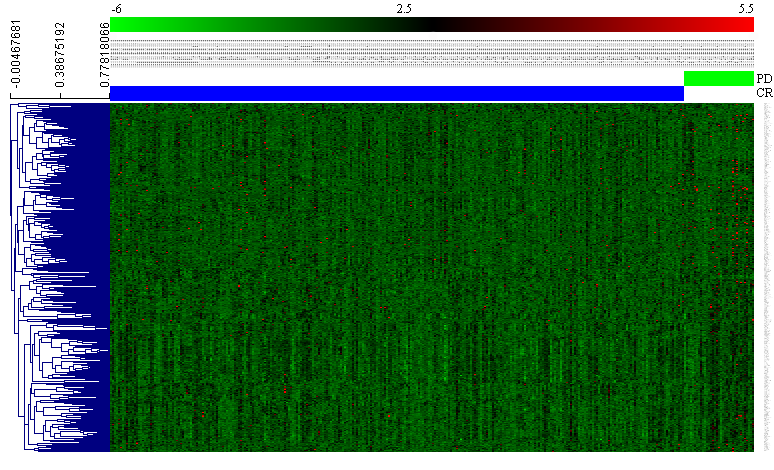

Supplement: Figure S1 — Heat map of 349-gene signature against 322 patients. This diagram shows the heat map of 349-gene signature against 322 patients, in which rows represent different genes in 349- gene signature and columns represent different patients. The blue bar above the heat map represents CR and green bar represents PD. (TIFF) [file pone.0052745.s010.tiff]

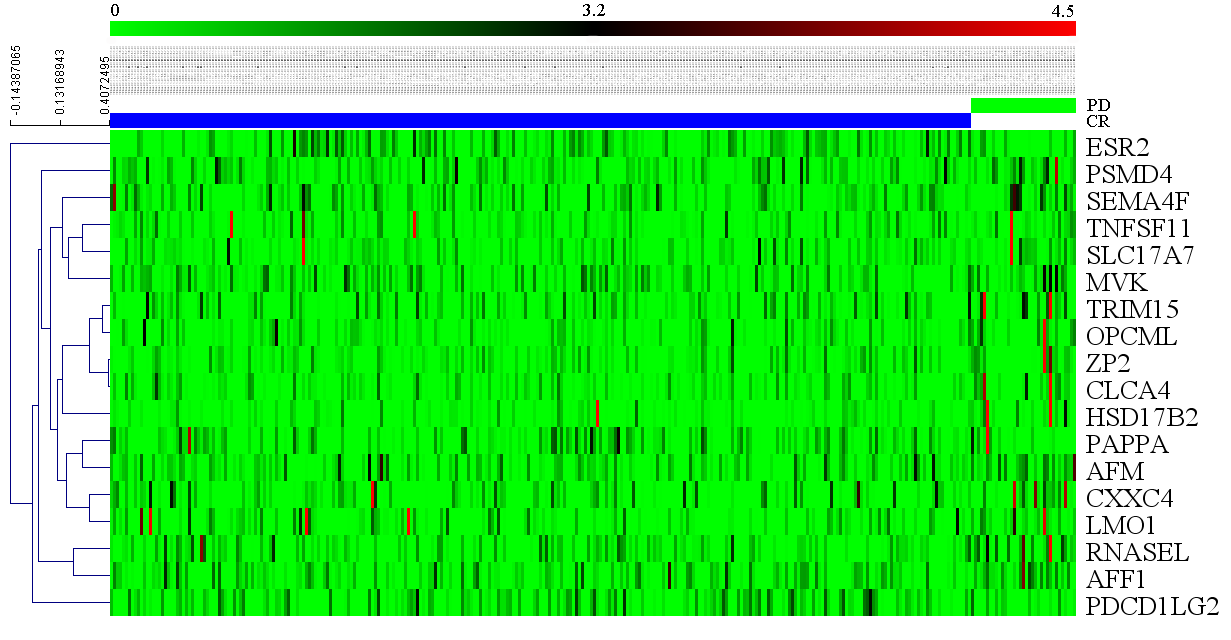

Supplement: Figure S2 — Heat map of 18-gene signature against 322 patients. This diagram shows the heat map of 18-gene signature against 322 patients, where rows represent different genes in 18-gene signature and columns represent different patients. The blue bar above the heat map represents CR and green bar represents PD. (TIFF) [file pone.0052745.s011.tiff]

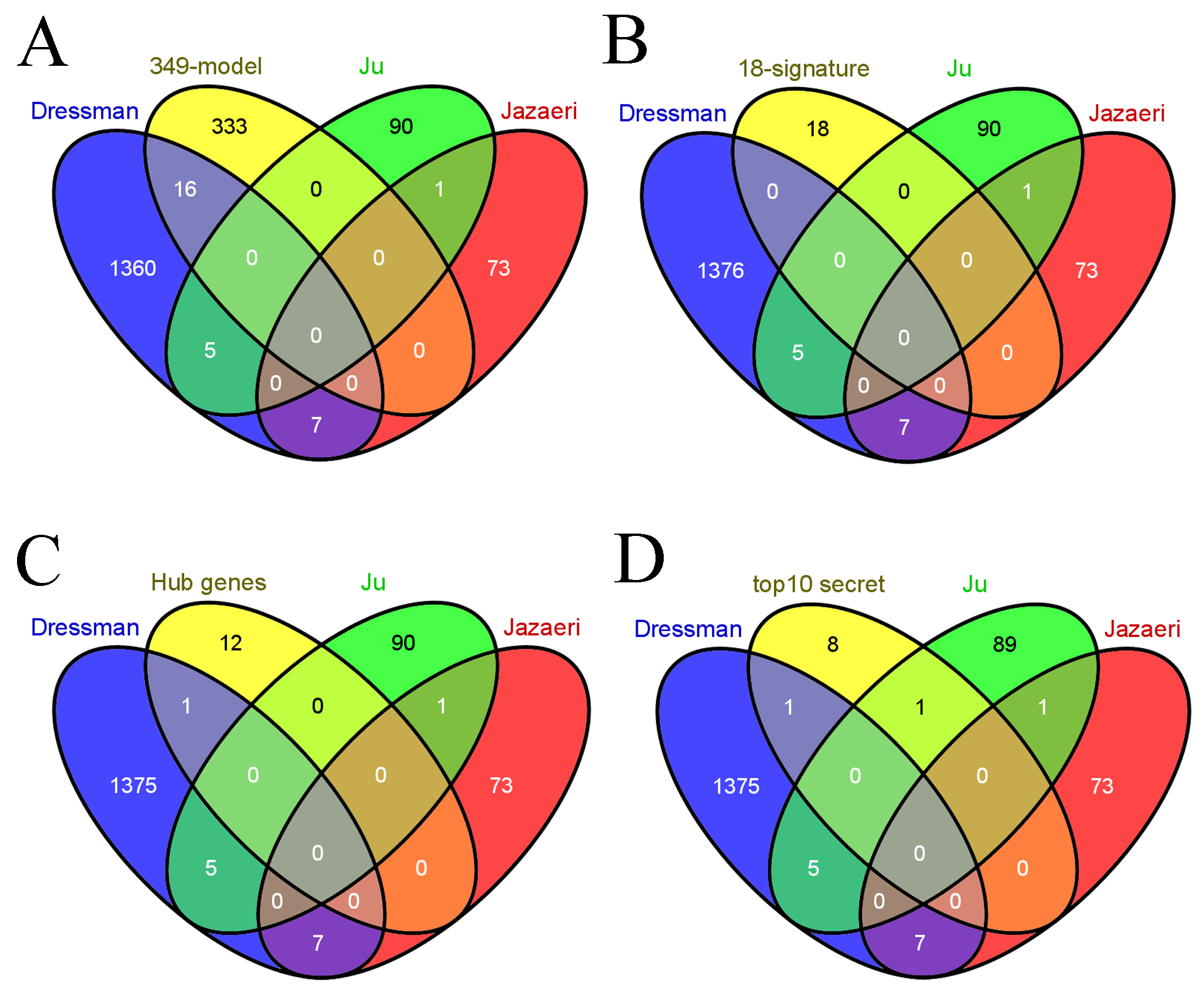

Supplement: Figure S3 — The Venn diagram showing the overlap between our signatures and genes from previous publications. The Venn diagram shows how much genes in the 349-gene model (A), 18-gene model (B), hub genes (C), top 10 serum biomarkers (D) are overlapped with 3 previous publications (Dressman et al., Ju et al. and Jazaeri et al.). (TIF) [file pone.0052745.s012.tif]

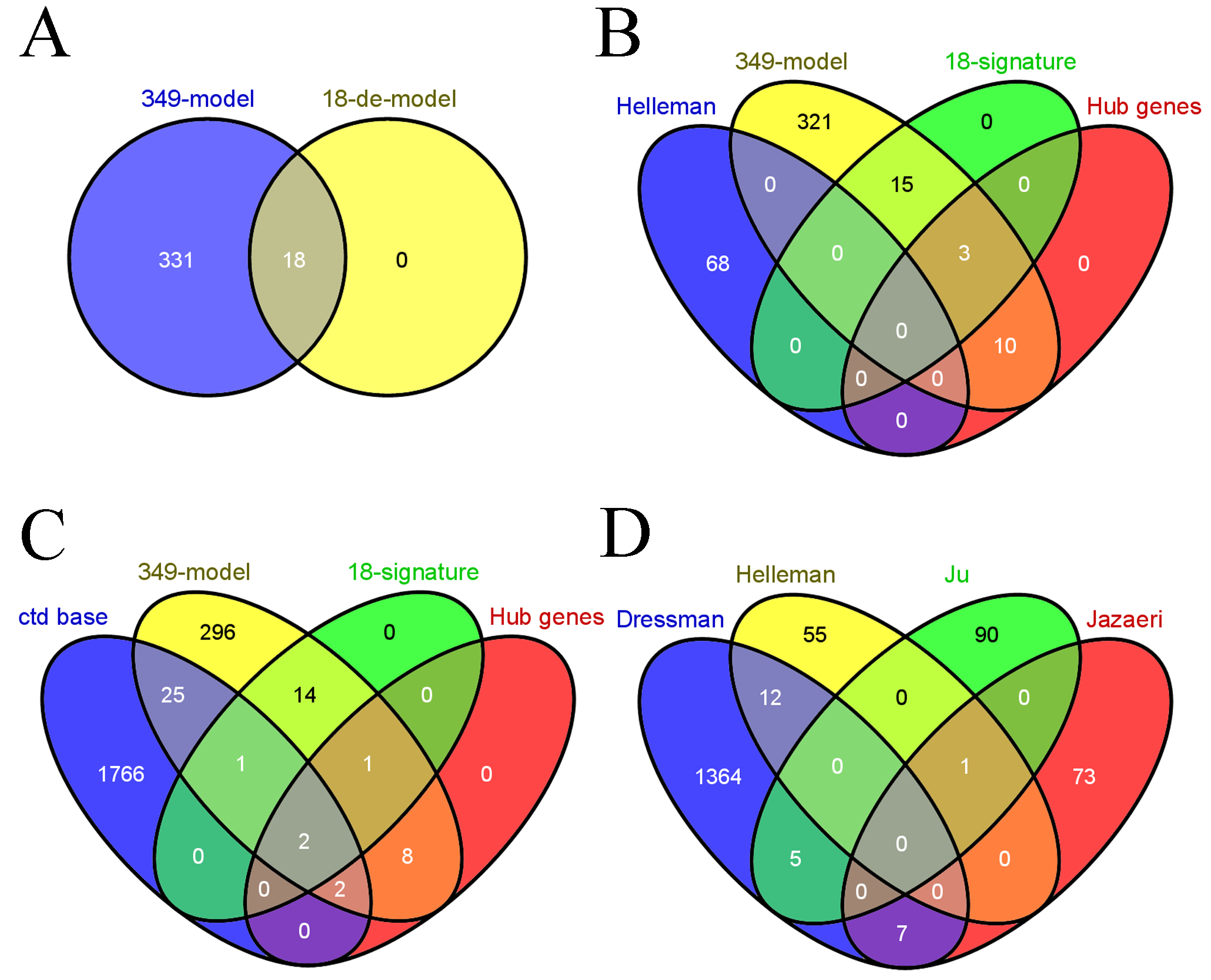

Supplement: Figure S4 — The Venn diagram showing the overlap among our signatures and other datasets & publications. (A) The Venn diagram shows genes in 18-gene signature are all belong to genes in 349-gene signature. (B) The Venn diagram shows there are no overlap between genes from Helleman et al. and genes in our findings (349-gene signature, 18-gene signature and hub genes). (TIF) [file pone.0052745.s013.tif]

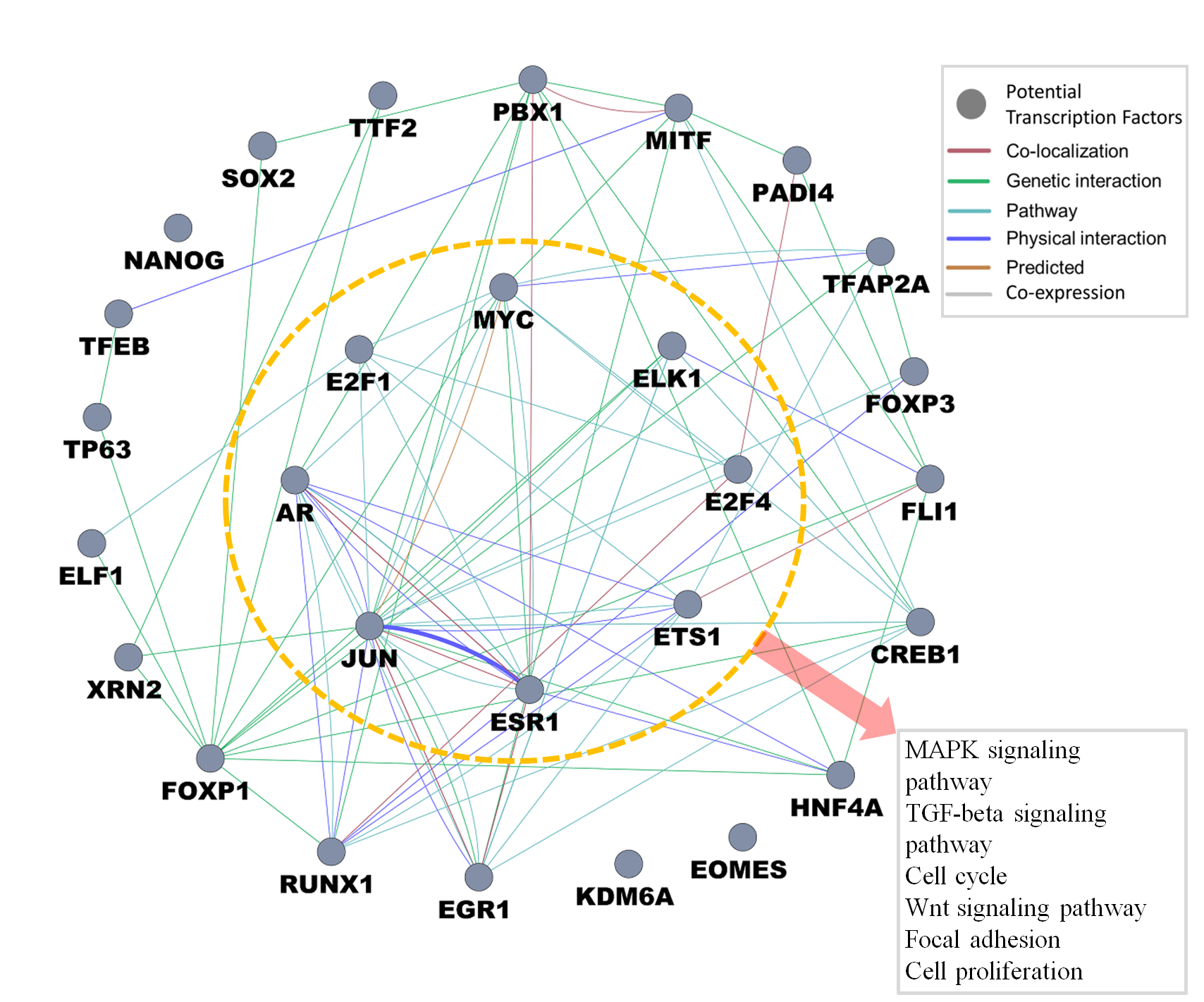

Supplement: Figure S5 — Transcription factor Interaction network and enriched pathways derived from the 349-gene model. Eight transcription factors circled in dot yellow line are enriched in TGF-beta, MAPK and Wnt signaling pathway (red arrow). (TIF) [file pone.0052745.s014.tif]

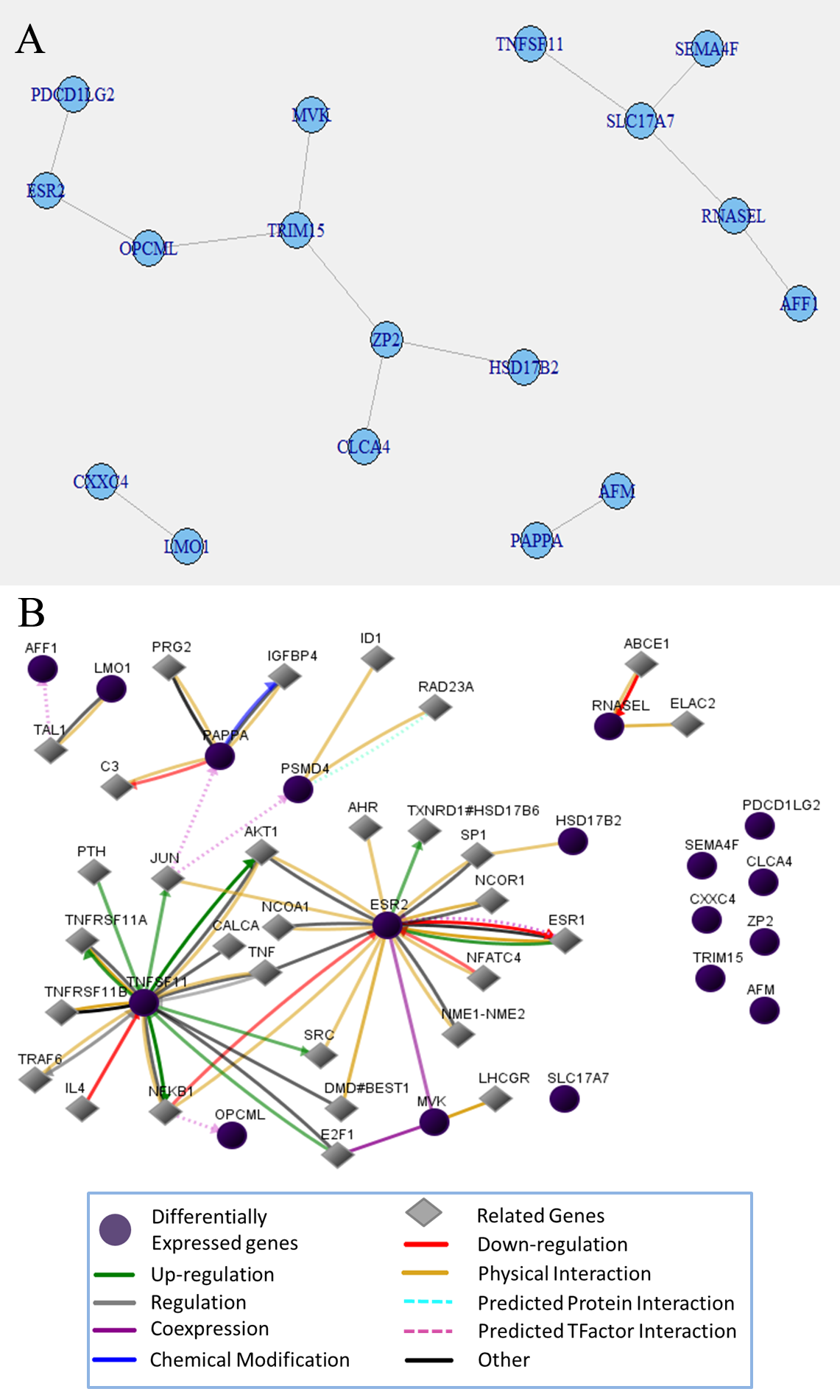

Supplement: Figure S6 — Gene-gene interaction network of 17 genes in the 18-gene model and Hub gene interaction network of 18 signature genes. (A) Gene-gene interaction network of 17 in the 18-gene model analyzed by C3NET. (B) Hub genes and neighboring genes of the 18 signature genes. (TIF) [file pone.0052745.s015.tif]

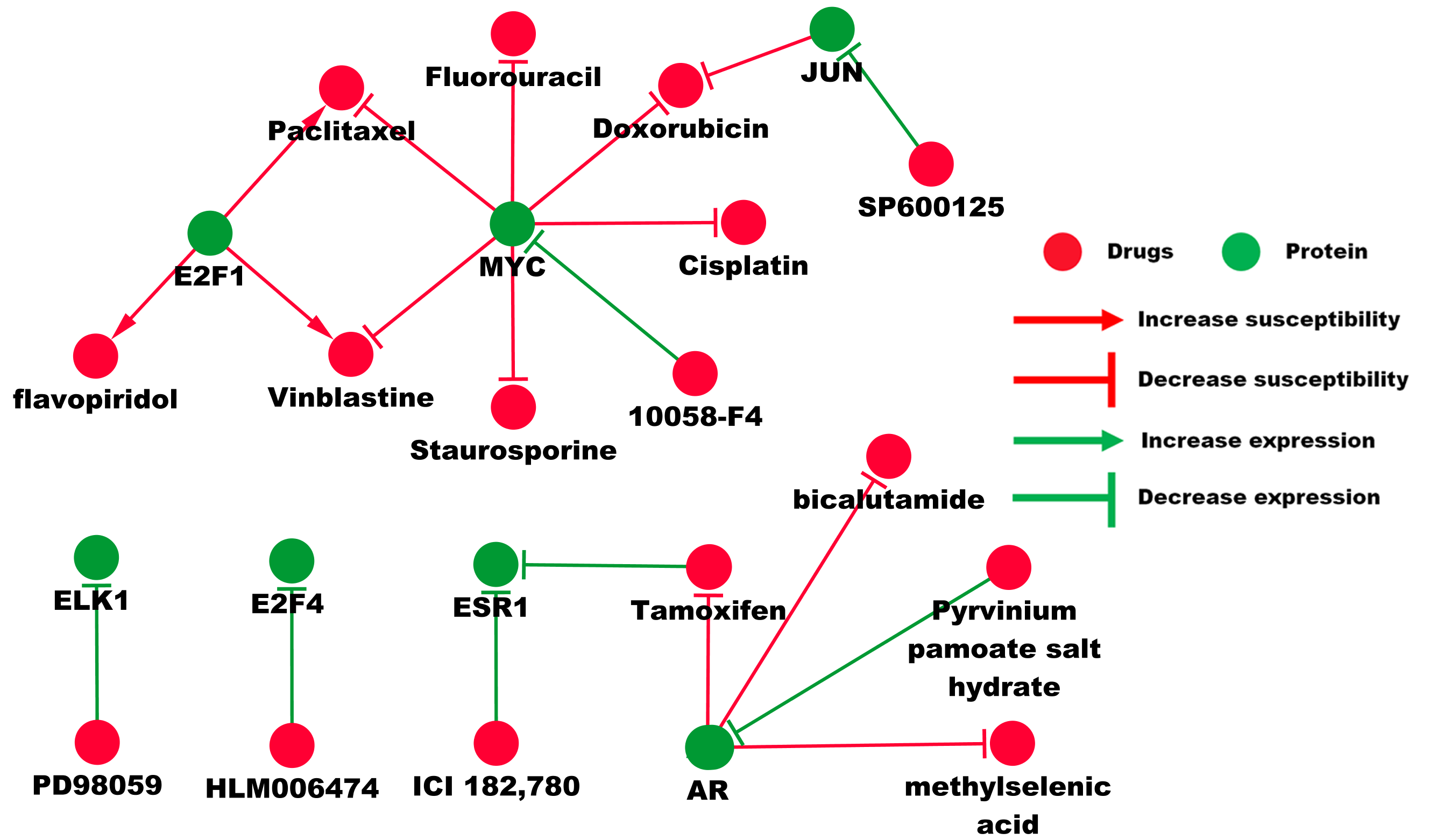

Supplement: Figure S7 — Transcription factor- drug Interaction network. This diagram shows how Transcription factors and drug are interacted. For example, MYC could decrease patient's susceptibility to Cisplatin, Fluorouracil and Doxorubicin, whereas 10058-F4 could decrease the expression of MYC. (TIF) [file pone.0052745.s016.tif]
